# BioVenn – a web application for the comparison and visualization of biological lists using area-proportional Venn diagrams

**DOI:** 10.1186/1471-2164-9-488

**Published:** 2008-10-16

**Authors:** Tim Hulsen, Jacob de Vlieg, Wynand Alkema

**Affiliations:** 1 Computational Drug Discovery (CDD), CMBI, NCMLS, Radboud University Nijmegen Medical Centre, P.O. Box 9101, 6500 HB Nijmegen, The Netherlands; 2 Molecular Design and Informatics, Schering-Plough, P.O. Box 20, 5340 BH Oss, The Netherlands

## Abstract

**Background:**

In many genomics projects, numerous lists containing biological identifiers are produced. Often it is useful to see the overlap between different lists, enabling researchers to quickly observe similarities and differences between the data sets they are analyzing. One of the most popular methods to visualize the overlap and differences between data sets is the Venn diagram: a diagram consisting of two or more circles in which each circle corresponds to a data set, and the overlap between the circles corresponds to the overlap between the data sets. Venn diagrams are especially useful when they are 'area-proportional' i.e. the sizes of the circles and the overlaps correspond to the sizes of the data sets. Currently there are no programs available that can create area-proportional Venn diagrams connected to a wide range of biological databases.

**Results:**

We designed a web application named BioVenn to summarize the overlap between two or three lists of identifiers, using area-proportional Venn diagrams. The user only needs to input these lists of identifiers in the textboxes and push the submit button. Parameters like colors and text size can be adjusted easily through the web interface. The position of the text can be adjusted by 'drag-and-drop' principle. The output Venn diagram can be shown as an SVG or PNG image embedded in the web application, or as a standalone SVG or PNG image. The latter option is useful for batch queries. Besides the Venn diagram, BioVenn outputs lists of identifiers for each of the resulting subsets. If an identifier is recognized as belonging to one of the supported biological databases, the output is linked to that database. Finally, BioVenn can map Affymetrix and EntrezGene identifiers to Ensembl genes.

**Conclusion:**

BioVenn is an easy-to-use web application to generate area-proportional Venn diagrams from lists of biological identifiers. It supports a wide range of identifiers from the most used biological databases currently available. Its implementation on the World Wide Web makes it available for use on any computer with internet connection, independent of operating system and without the need to install programs locally. BioVenn is freely accessible at .

## Background

In many genomics projects and other projects handling large amounts of biological data, various lists containing biological identifiers are produced, corresponding to e.g. sets of genes regulated under different treatments. Often, it is useful to see the overlap between these lists. This enables researchers to quickly observe similarities and differences between the data sets they are analyzing. One of the most popular methods to visualize the overlap and differences between data sets is the Venn diagram, named by its inventor John Venn [1]. A large number of different types of Venn diagrams exist, the most popular being the three-circle Venn diagram, used to visualize the overlap between three data sets. In such a diagram, the size of the circle can be used to represent the size of the corresponding data set. This is called an area-proportional Venn diagram [2]. Venn diagrams have been used recently to visualize gene lists [3,4]. However, these applications generate diagrams with circles of equal size.

There are some computer programs available that generate area-proportional Venn Diagrams, either as rectangles [5] or as polygons [6]. Drawback of these programs is that they need to be downloaded and run locally, limiting their use by a wide community. There is also the Google Chart API [7], which can generate circular, area-proportional Venn Diagrams, but can only have three numbers as input, and cannot do any calculations to obtain these three numbers. There is currently no web application available that can generate circular, area-proportional Venn diagrams connected to a wide range of biological databases, and can map different kinds of IDs to genes. In this article, we present a web application named BioVenn which can generate circular, area-proportional Venn diagrams just by entering two or three lists of biological IDs. IDs that can be recognized by BioVenn as belonging to a certain database, are linked to that database. BioVenn currently supports cross-references to Affymetrix [8], COG [9], Ensembl [10], EntrezGene [11], Gene Ontology [12], InterPro [13], IPI [14], KEGG Pathway [15], KOG [16], PhyloPat [17] and RefSeq [18]. BioVenn is based on a previous version [19], which has been used in several scientific publications to visualize sets and their overlapping areas [20-22].

## Methods

### Construction of the Venn diagrams

**Figure 1:**
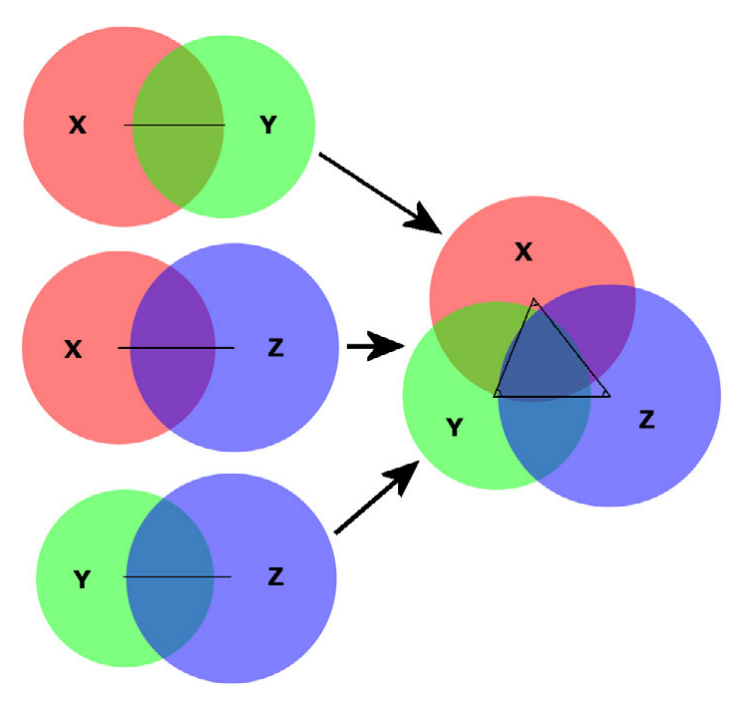
**Construction of a three-circle BioVenn diagram**. The method for generating a three-circle BioVenn diagram. The distance between the centers of each pair of circles is calculated, taking into account the size and overlap of the circles. Each pair of circles is put together using these distances. Then the three circles are put together, generating a three-circle diagram with not only two-circle overlaps but also a three-circle overlap.

The PHP script that calculates the proportions of the Venn diagram, including the overlap between the circles, was written using information from the Wolfram MathWorld website [23,24]. It calculates the distance between the centers of each pair of circles (X-Y, X-Z and Y-Z), taking into account the size of each circle and the size of the overlap between the two circles. Then the three circles are put together by adjusting the angles between the three circles (Fig. 1), which are 60° for circles of the same size.

### The input page

**Figure 2:**
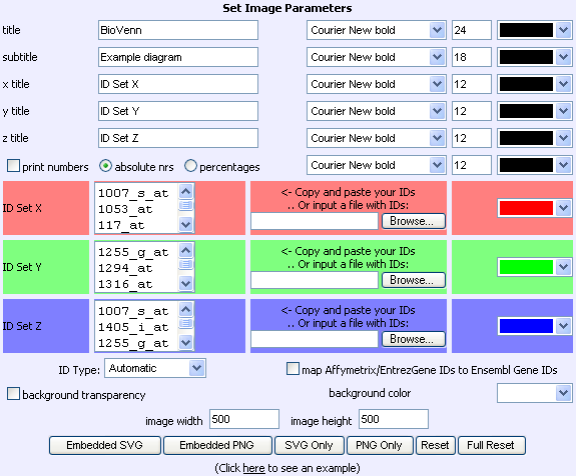
**The BioVenn input page**. The BioVenn input page, with an example of three lists of Affymetrix probe identifiers.

The input page (Fig. 2) offers some parameters for easy input of the data, as well as some formatting options. A title and subtitle can be entered, as well as their font type and font size. Each of the ID sets can be given their own name, so that the user can immediately see which part of the output corresponds to which input list. The user can also choose to print the numbers of IDs in the Venn diagram, as either absolute numbers or percentages of the total number. All of these textual parameters can be given their own color using a dropdown menu containing eighteen colors.

The second part of the input page has two input options for each of the three ID sets: a copy-and-paste input field and a file input field. BioVenn will automatically remove any duplicate IDs. The default colors of sets 1, 2 and 3 are red, green and blue, but the user can choose to select different colors, again by using a dropdown menu. If one of the three ID set input fields is left empty, BioVenn will generate a diagram of only two circles.

In the lower part of the input page, the user can pick a background color, or choose for background transparency. The user can also change the total width and height of the output SVG image. The "Create Embedded SVG" button generates an SVG image embedded in the HTML page, whereas the "Create SVG Only" button sends the SVG image directly to the browser. The latter option is especially useful for batch queries. Instead of SVG, the user can choose to display the Venn diagram as a (non-clickable) PNG image. The "Reset" button puts all parameters back to the current image, and the "Full Reset" button puts them back to default. Finally, there is a link to an example generated by a small number of Affymetrix IDs, for those who want to see a sample Venn diagram immediately. This link also shows how a Venn diagram can be created by entering the ID lists (plus titles and other parameters) in the URL, e.g. . IDs are recognized automatically where possible, but the user can also choose from a dropdown list which type of IDs is used as input. BioVenn offers an optional mapping from Affymetrix IDs and EntrezGene IDs to Ensembl Gene IDs (version 50) for the species *H. sapiens*, *M. musculus *and *R. norvegicus*, for researchers that want to do a gene-based comparison from expression data.

## Results & Discussion

### The output Venn diagram

**Figure 3:**
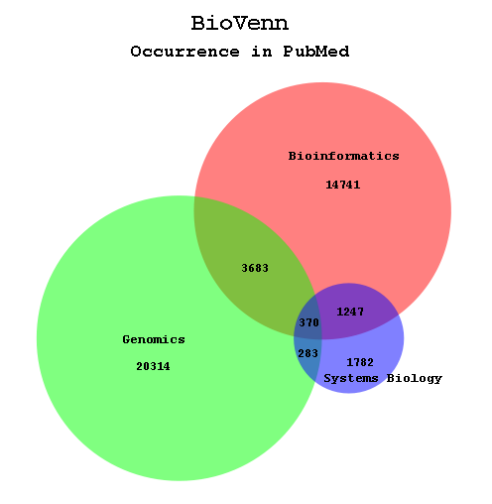
**Example BioVenn diagram**. The BioVenn diagram resulting from a PubMed comparison of the terms 'Bioinformatics', 'Genomics', and 'Systems Biology'.

The BioVenn output (Fig. 3) consists of an SVG or PNG image of two or three circles, in which each circle represents one of the ID sets used as input. The size of the circle corresponds with the number of unique IDs in that specific set. The overlap of each two circles also corresponds with the number of IDs belonging to both of the sets represented by these circles. The overlap between all three circles (XYZ overlap) is also shown, but due to mathematical reasons (more degrees of freedom are needed) the size of this overlap cannot always correspond exactly with the number it represents, as noticed by several mathematics studies [2,25]. However, in many cases creating the right two-circle overlaps will also give the correct three-circle overlap. In the SVG image, the position of the titles, numbers and percentages (if enabled) can be adjusted by drag-and-drop. When using one of the newer SVG plugins, users have some extra options, such as zooming in and out or moving the diagram around.

### Image statistics

**Figure 4:**
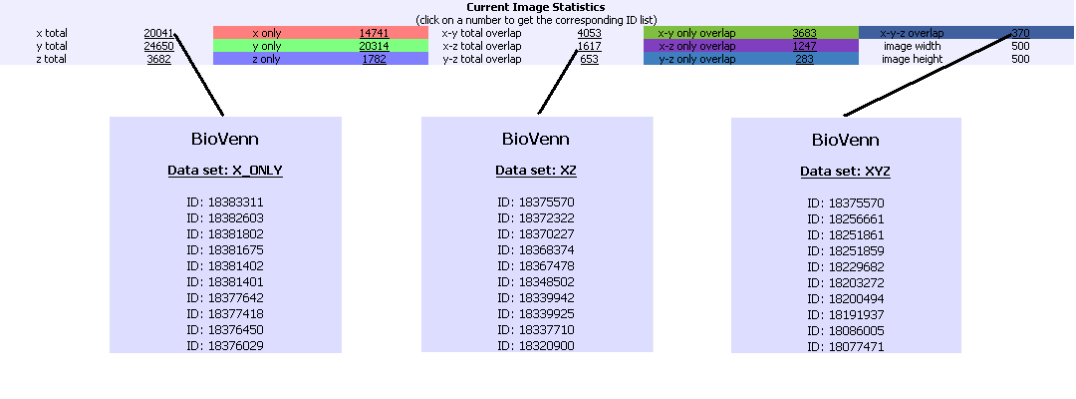
**Current image statistics**. The image statistics page belonging to the example from figure 3.

Below the SVG or PNG image, the numbers belonging to the currently shown image are displayed (Fig. 4). Clicking on one of these numbers opens a popup window with the corresponding list of IDs. If the type of ID is recognized as (or defined by the user as) Affymetrix, COG, Ensembl, EntrezGene, Gene Ontology, InterPro, IPI, KEGG Pathway, KOG, PhyloPat or RefSeq ID, the ID will be linked to the database page with more information about that ID.

## Conclusion

BioVenn is an easy-to-use web application to generate area-proportional Venn diagrams from lists of biological identifiers. It supports a wide range of identifiers from the most used biological databases currently available. Its implementation on the World Wide Web makes it available for use on any computer with internet connection, independent of operating system and without the need to install programs locally.

## Availability & requirements

BioVenn is freely available at  and has been tested extensively in Internet Explorer and Mozilla Firefox. For browsers that do not have native SVG support, a free SVG plugin can be downloaded from either  (Adobe SVG Viewer) or  (RENESIS Player).

## Abbreviations

COG: Clusters of Orthologous Groups of proteins; IPI: International Protein Index; KEGG: Kyoto Encyclopedia of Genes and Genomes; KOG: euKaryotic Orthologous Groups of proteins; SVG: Scalable Vector Graphics.

## Authors' contributions

TH participated in the design of the study, built the application, and drafted the manuscript

JdV participated in the design of the study

WA participated in the design of the study and helped to draft the manuscript
